# Current Knowledge about Nonclassical Monocytes in Patients with Multiple Sclerosis, a Systematic Review

**DOI:** 10.3390/ijms25137372

**Published:** 2024-07-05

**Authors:** Borros Arneth

**Affiliations:** 1 Institute of Laboratory Medicine and Pathobiochemistry, Hospital of the Universities of Giessen and Marburg, UKGM, Philipps University Marburg, Baldingerst 1, 35043 Marburg, Germany; borros.arneth@staff.uni-marburg.de; 2 Institute of Laboratory Medicine and Pathobiochemistry, Hospital of the Universities of Giessen and Marburg, UKGM, Justus Liebig University Giessen, Feulgenstr 12, 35392 Giessen, Germany

**Keywords:** classical-monocytes, non-classical monocytes, intermediate monocytes, multiple sclerosis

## Abstract

Monocytes play a critical role in the initiation and progression of multiple sclerosis (MS). Recent research indicates the importance of considering the roles of monocytes in the management of MS and the development of effective interventions. This systematic review examined published research on the roles of nonclassical monocytes in MS and how they influence disease management. Reputable databases, such as PubMed, EMBASE, Cochrane, and Google Scholar, were searched for relevant studies on the influence of monocytes on MS. The search focused on studies on humans and patients with experimental autoimmune encephalomyelitis (EAE) published between 2014 and 2024 to provide insights into the study topic. Fourteen articles that examined the role of monocytes in MS were identified; the findings reported in these articles revealed that nonclassical monocytes could act as MS biomarkers, aid in the development of therapeutic interventions, reveal disease pathology, and improve approaches for monitoring disease progression. This review provides support for the consideration of monocytes when researching effective diagnostics, therapeutic interventions, and procedures for managing MS pathophysiology. These findings may guide future research aimed at gaining further insights into the role of monocytes in MS.

## 1. Introduction

Multiple sclerosis (MS) is an autoimmune condition in which the central nervous system exhibits demyelination and chronic inflammation, which lead to adverse neurological symptoms. MS pathology involves complex immune cell interactions that affect disease initiation, progression, and modulation. Although multiple studies have examined the roles of immune cells in MS pathogenesis, the functions of monocyte subsets, specifically nonclassical monocytes (NCMs), remain unclear [1]. Previous studies have reported that NCMs are anti-inflammatory and homeostatic and act as the first line of defense against pathogens [2,3,4,5,6]. However, other studies have suggested that NCMs have proinflammatory properties [5], can infiltrate the central nervous system [7], can present antigens [8], can mediate tissue damage [9], and are potential therapeutic targets [10]. The multifaceted perspective on the roles of NCMs in MS pathogenesis presents an opportunity to explore many therapeutic approaches to achieve optimal patient care. This systematic review provides up-to-date insights into the role of NCMs in the initiation and progression of MS, as well as in modulating related immune responses.

What are classical and nonclassical monocytes? This classification is performed via flow cytometry. The definitions are as follows:

CD14^+^CD16^++^ monocytes are called nonclassical monocytes.

CD14^++^CD16^−^ monocytes are called classical monocytes.

CD14^++^CD16^+^ monocytes are called intermediate monocytes.

## 2. Methods

This systematic review examined up-to-date information on how NCMs influence MS. It followed the Preferred Reporting Items for Systematic Reviews and Meta-Analyses (PRISMA) guidelines to collect, analyze, and report on the relevant literature [11,12]. The review addresses the PICOT question: “*In patients diagnosed with multiple sclerosis (Population), does the involvement of nonclassical monocytes (Intervention) compared to that of classical and intermediate monocytes (Comparison) have a significant impact on the inflammatory processes within the central nervous system, contribute to demyelination and neurodegeneration, and influence tissue repair and the resolution of inflammation (Outcome), as reported in studies published within the last 10 years (Time)*?”

A literature search of the PubMed, EMBASE, Cochrane, and Google Scholar databases was performed with the following search terms: {(“nonclassical monocytes”/“CD14^+^CD16^++^”/“patrolling monocytes”) + (“multiple sclerosis”/“MS”) + (“inflammatory processes”/“demyelination”/“neurodegeneration”/“tissue repair”) + (“classical monocytes”/“monocyte subsets”)}. The study has been registered under https://osf.io/d352v/ (accessed on 1 July 2024, Open Science Framework).

### 2.1. Inclusion and Exclusion Criteria

The influence of NCMs on MS has been documented in many studies, and strategic inclusion and exclusion criteria were needed to find the most relevant articles. Sanfilippo et al. stated that inclusion and exclusion criteria offer guidelines for achieving the desired outcome [13]. The inclusion and exclusion criteria in this review were related to the nature of the research studies, participants, interventions, and trial periods. This systematic review included original research articles on MS involving animal or human participants published within the last 10 years and written in English. The exclusion criteria were review articles or nonoriginal research articles, articles on studies lacking human or animal participants, articles published more than 10 years ago, and articles not written in English.

This review also identified the most important articles that uniquely contributed to the field. These are studies that offer quality results through insightful information on the role of nonclassical monocytes in MS patients. The researcher identified the most important articles by examining their relevance to the research topic, contribution to knowledge, methodological rigor, novelty and innovation, and the scope and breadth of their findings. With respect to defining the relevance of the study, the researcher sought articles with direct relevance to the roles of monocytes in the pathogenesis, progression, diagnosis, and treatment of MS. For contributing to knowledge, the researcher sought studies that significantly contributed to advancing knowledge on the selected topic and categorized them among the most important. The category of methodological rigor focused on articles with robust methods, such as large sample sizes, properly designed experiments, and in-depth data analysis. Articles with methodological rigor offer abundant information on the role of monocytes in MS.

The researcher applied novelty and innovation criteria to identify papers with novel concepts, methodologies, or findings on the topic that offer unique insights or perspectives. Finally, the researcher considered important articles based on the scope and breadth of the findings. The selected articles must cover a broad range of aspects of monocytes in MS. Critical areas of analysis include their roles in inflammation, immune modulation, diagnosis, and therapeutic interventions. The above criteria provide a blueprint for identifying the articles with the greatest impact and that offer affluent knowledge that significantly challenges the existing knowledge on the subject. Subsequent studies can rely on information from these articles to examine and integrate new areas of knowledge in research on the issue.

**Figure 1:**
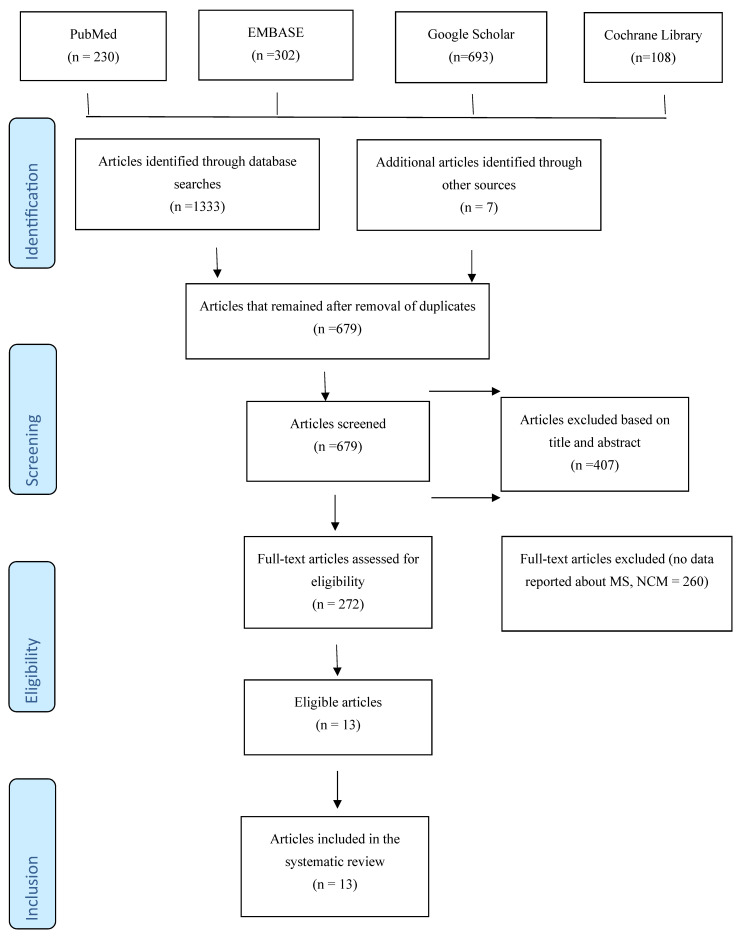
PRISMA flowchart.

Figure 1 shows the PRISMA flow chart for the search algorithm used here.

### 2.2. Limitations of the Study

Since the review was carried out by only one person, there may have been a certain bias. The author tried to make the review as objective and open as possible. However, it cannot be entirely ruled out that bias occurred.

## 3. Results

A literature search of the indicated databases identified 1333 articles, and Google searches identified 7 additional journal articles. The articles were screened, and 654 duplicates were removed, leaving 679 articles. The abstracts and titles of the remaining articles were examined to determine their relevance to the research objective; a total of 407 articles were removed, leaving 270 articles. The remaining articles were further screened to eliminate those that lacked full text or sufficient data to ensure accuracy; 256 articles were eliminated, leaving 13 articles for inclusion in the systematic review.

**Figure 2:**
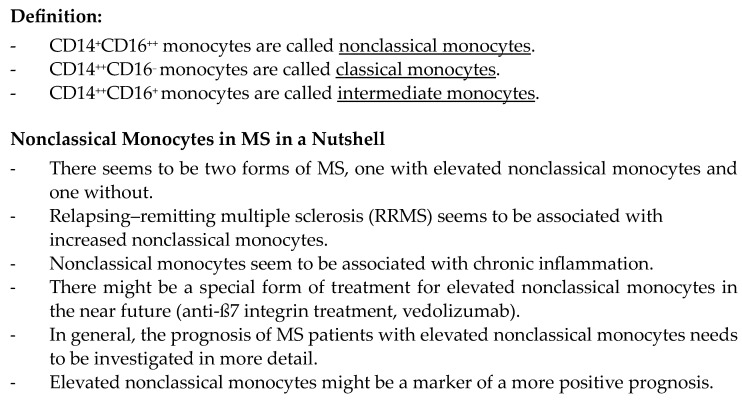
Take-home messages.

The analysis further identified the ten most important findings and articles, which comprised research that examined the issue in depth and provided new knowledge on the study subject. These important findings represent milestones in understanding the role of nonclassical monocytes in MS patients. Figure 2 shows the most important take home messages found through this review.

Table 1 gives the details of the selected studies.

## 4. Discussion

The levels of classical, nonclassical, and intermediate monocytes in MS patients reflect the dynamic nature of the immune response. The dysregulation of monocyte subsets supports the potential of these cells as biomarkers for disease initiation and progression and offers potential targets for the development of effective and lasting interventions [9,14,25]. Multiple studies have provided insights into the relationship between monocytes and MS. The following discussion examines what is known about monocytes and MS, the location of monocytes within MS patients, and the roles of monocytes in animal models of MS.

### 4.1. Current Knowledge

Previous studies have offered distinct perspectives on the role of classical, intermediate, and nonclassical monocytes in MS. Monocyte populations have different functions in the central nervous system (CNS) and peripheral immune system pathology during neuroinflammation [26]. Monocytes are generated from pluripotent stem cells in the bone marrow through promonocyte division [27]. Monocytes then enter the circulation as circulating and marginating pools, with each pool comprising approximately 50% of the monocytes circulating in blood vessels [28,29]. The multifaceted roles of classical, intermediate, and nonclassical monocytes in MS highlight the complex interplay among MS pathophysiology, immune cells, the CNS, and the peripheral immune system. Notably, understanding monocyte generation and circulation has revealed critical aspects of MS pathology.

Classical monocytes exhibit distinct behaviors that trigger immune activation and tissue maintenance. Monocytes relocate to the CNS and transform into antigen-presenting cells during disease progression. Classical monocytes infiltrate tissues and produce inflammatory cytokines; these cells then differentiate into macrophages [30]. Classical monocytes also remove microorganisms and dying cells from the body [31,32]. Although classical monocytes are known to play an inflammatory role, recent studies have revealed that these monocytes are primarily phagocytic rather than inflammatory. Multiple studies have revealed the abundance of classical monocytes in inflamed regions in the context of MS, highlighting their phagocytic and inflammatory functions.

NCMs have anti-inflammatory and proinflammatory effects that define their functions in MS. These cells patrol the peripheral and central nervous systems to identify any damage. NCMs differentiate into anti-inflammatory macrophages to repair damaged tissues and under steady-state conditions, into tissue-resident macrophages [33]. NCMs influence MS by entering the peripheral circulation and triggering proinflammatory and anti-inflammatory effects [34]. Some studies have shown that NCMs are proinflammatory due to their role in promoting inflammation, crossing the blood–brain barrier, and triggering immune cell infiltration into the CNS [35]. The severity of MS and its clinical manifestations are determined by the presence and quantity of NCMs in the peripheral blood and CNS.

### 4.2. The Role of Monocytes in Clinical Disease

The synthesized literature offers a broad perspective on the role of NCMs in MS. Recent research offers new perspectives on how NCMs influence MS and possible avenues for advanced treatment approaches [36]. The core themes in the literature include increased monocyte subsets in MS patients, inflammatory responses and cytokine dysregulation, potential therapeutic interventions, monitoring disease activity and diagnosis, and immunomodulation and anti-inflammatory parameters.

The abundant monocyte subsets in MS patients offer an approach for a more effective and accurate disease diagnosis. The published articles revealed that NCMs are present in large numbers in MS patients. Fischer et al. reported that the number of classical CD14^++^CD16^−^ monocytes is higher and the number of nonclassical CD14^+^CD16^++^ monocytes is lower in MS patients than in healthy patients [15]. Khater et al. also reported that MS patients exhibit reduced levels of monocyte subsets during the relapse and remission stages of the disease [16]. Gjelstrup et al. observed a tendency for an expanded population of NCMs in MS patients [17]. This finding provides valuable insights into the role of monocytes in the pathogenesis of MS and reveals that nonclassical monocytes could be a therapeutic approach for modulating neuroinflammation. Rossi et al. also observed an increase in the population of NCMs in MS patients [34]. Other recent studies [2,37,38,39,40] have shown increases in monocyte subsets, such as the NCM subset. In MS patients, the NCM population increases, and these NCMs patrol and remove debris from the vasculature [41] and participate in other inflammatory conditions that contribute to disease progression. Increased numbers of NCMs represent a promising focus for advanced research on effective diagnostic procedures to support understanding of disease initiation, progression, and remission.

The monocyte inflammatory response and cytokine dysregulation reveal the immunopathogenesis of MS and its complex immune dysregulation. Studies have revealed that MS patients exhibit inconsistent distributions of monocyte subsets, confirming the dynamic nature of this disease. MS patients exhibit altered monocyte inflammatory responses and dysregulated anti-inflammatory and inflammatory cytokine levels. This observation suggests the possibility of monocyte-mediated inflammation as a therapeutic intervention in MS, highlighting that monocyte modulation could pave the way for novel treatments [18,42,43]. Other studies revealed differences in the levels of NCMs between patients with MS and those with neuromyelitis optica spectrum disorder. Waschbisch et al. reported that in MS patients, CD16^+^ monocyte function was reduced in the blood but increased in the cerebrospinal fluid [19]. These findings highlight the importance of trafficking immune cells across the blood–brain barrier and indicate that monocyte migration is a promising therapeutic approach for modulating neuroinflammation in MS. MS patients exhibit elevated levels of CD14^+^CD16^+^ monocytes, which produce IL-6 and IL-10 [44,45]. These findings offer insight into MS pathogenesis and suggest approaches for specific diagnostic strategies and therapeutic interventions for MS. The decreased function of CD16^+^ monocytes in the blood and their increased function in the cerebrospinal fluid reveal a possible cell migration path into the CNS during disease progression [6,46,47]. Further research on monocyte behavior is needed to determine the role of these cells in MS and to develop effective diagnostic and therapeutic interventions.

These studies reveal the therapeutic potential of modulating NCM function. The behaviors of monocyte subsets have diverse implications for MS treatment. Fischer et al. reported that glucocorticoid treatment triggered the generation of an anti-inflammatory phenotype, which caused monocyte migration into the CNS and suppressed pathogenic immune responses [15]. These findings suggest that researchers could use monocyte polarization to mitigate neuroinflammation and further improve clinical outcomes. Maleki et al. reported that administering muramyl dipeptide (MDP) to mice delayed experimental autoimmune encephalitis (EAE) onset and reduced leucocyte infiltration [20]. Research targeting and inhibiting monocyte-mediated inflammation is a promising therapeutic strategy to prevent or delay neuroinflammation effects or delay MS onset. Kapate et al. reported that NCMs can carry particles (“backpacks”) loaded with IL-4 and dexamethasone, triggering anti-inflammatory effects that relieve MS symptoms [21]. Moreover, Beyers reported that clozapine induces an anti-inflammatory effect by increasing the number of NCMs and preventing the expression of proinflammatory cytokines (IL-6, IL-1β, and TNFα) [48]. As such, clozapine treatment decreases the levels of cytokines that influence MS pathology [49]. Radandish reported that altering CNS homeostasis triggers the excretion of tissue-resident macrophages in the parenchyma, improving symptoms [50]. In a study by Meyer-Arndt, the administration of lipoic acid inhibited monocyte secretion of the cytokines responsible for MS [22]. Adriani reported that treating MS patients with IFN-β triggered the production of CD14^+^ monocytes, thus increasing the population of proinflammatory monocytes [23]. The above findings show that modulating NCM function offers a basis for the development of treatments to alleviate MS symptoms in both the initial and progressive phases.

Researchers can utilize NCMs to diagnose and monitor MS disease activity. Studies have revealed that the population distribution of monocyte subsets in MS patients corresponds to distinct disease stages and can provide insight into disease prognosis and pathology. Khater et al. reported that during MS relapse and remission, the number of NCMs increases significantly [16]. These results revealed the dynamic changes in the monocyte population in MS patients, revealing the need to monitor monocyte subset changes to assess disease activity and guide treatment options.

On the other hand, Carstensen et al. reported that NCM numbers are reduced in patients with incipient MS, and they linked HERV expression to inflammatory activation [9]. The above findings suggest a possible role of nonclassical monocytes as biomarkers for incipient MS, emphasizing the need for more research into HERV expression in MS patients. Haschka et al. also reported that patients with untreated relapsing–remitting MS have reduced levels of NCMs, revealing an association between alterations in the levels of monocyte subsets and MS activity [10,22]. Clearly, there is a need to understand monocyte dynamics better and to determine how changes in monocyte subsets can reveal disease progression and treatment. By comparing different disease phases and examining disease activity, Ajami found that NCMs may be potential biomarkers for monitoring MS [1]. Monteiro also observed a reduction in plasmacytoid dendritic cells (pDCs) and myeloid dendritic cells (mDCs) during MS remission and the return to normalcy, and the frequency of NCMs also decreased [24]. The immunological changes that occur during MS relapse and remission suggest that dendritic cell subsets can enable researchers to assess disease progression and treatment responses. 

In the context of active disease, monocyte activation and recruitment occur in the CNS, causing neuroinflammation and tissue damage. Increased monocyte activity contributes to the relapse of symptoms. During disease progression, monocyte activation is sustained, causing chronic inflammation and progressive neurodegeneration. At remission, disease activity slows, reducing inflammatory responses. The above studies collectively provide support for research focused on monocyte-related therapeutic interventions in MS; researchers can use the above information as the basis for research into effective MS interventions.

Immune modulation and anti-inflammatory parameters are potential therapeutic targets for MS. Režić Mužinić et al. reported that classical monocytes express CD40, which has proinflammatory effects, and CD192, which has anti-inflammatory effects [51]. The decreased expression of surface receptors in MS patients with both normal and altered motor symptoms points to the potential role of immune cell signaling dysregulation and how it causes neurological impairment and the progression of MS. Bai et al. postulated that NCMs exhibit proinflammatory activity by supporting inflammatory cells during infections [52,53]. NCMs can migrate quickly into the brain and CNS when infection occurs [54,55]. The anti-inflammatory characteristics of classical and nonclassical monocytes highlight the complexity of immune responses [56]. Notably, Monaghan et al. reported that genes encoding proinflammatory cytokines are expressed in the CNS, suggesting that monocytes play a role in MS pathogenesis [57]. These findings pave the way for the establishment of interventions that address the robust activity of pro- and anti-inflammatory pathways, leading to more practical and tailored MS treatments.

This synthesis of studies on the role of NCMs in MS reveals distinct perspectives on their influence, translating into substantial insights for improvements in patient care and treatment. The increased presence of NCMs offers a promising basis for research to improve MS diagnosis and treatment consistent with the immunological profile of this disease. Monocytes exhibit dynamic inflammatory and anti-inflammatory responses, necessitating tailored diagnostics and therapeutic interventions. The therapeutic potential of NCMs also represents a significant step toward developing effective approaches for alleviating symptoms at distinct stages of MS. 

Studies have also identified NCMs as possible diagnostic biomarkers for monitoring disease activity based on an improved understanding of complex MS pathology. Increased understanding of the interplay between classical and nonclassical monocytes has revealed CD40 and CD192 as markers and potential therapeutic targets. Knowledge of the proinflammatory activity of NCMs highlights the complex immune responses at work. These findings provide a foundation for exploring the roles of NCMs in MS and customizing therapeutic approaches for disease management at the initial and progressive stages. 

In subsequent studies, the proportions of nonclassical, intermediate, and classical monocytes should be measured to clarify their distinct roles in inflammation and the immune response in MS. The characteristics of each subset offer useful information on the immune response and inflammation. For instance, increased levels of classical monocytes indicate tissue damage or ongoing inflammation, and changes in NCMs indicate alterations in immune surveillance and tissue repair in MS patients. Intermediate monocytes may reveal changes in inflammatory stimuli and immune activation in MS patients. Monocyte subset measurements reveal immune dysregulation in MS and, thus, represent biomarkers for disease activity. Monocyte subsets can be used to identify MS initiation, progression, and remission. Although monocyte subsets offer valuable information about immune responses in MS, clinical researchers cannot use these subsets to directly assess the treatment response, predict future relapses, or guide treatment decisions. Clinical researchers should adopt comprehensive approaches that integrate diverse parameters to manage MS effectively. Table 2 gives the 10 key papers (most important papers) in the field while Table 3 gives the most important findings in the field.

### 4.3. Practical Use

For a long time, classical monocytes have been viewed as critical for the initial inflammatory response, and nonclassical monocytes have been viewed as anti-inflammatory and protective, as they seemed to maintain vascular homeostasis. However, according to novel data, the function of these cells seems to be different in chronic diseases in humans as in human MS.

The development of the monocytes in humans during the MS disease starts with the classical monocytes; then, they transform into the intermediate monocytes, and the process ends with the nonclassical monocytes. This development goes hand in hand with an increasing duration of the MS-disease and with an increasing chronicity of the MS-disease. Scientists nowadays assume that nonclassical monocytes are present in significantly higher amounts in chronic MS patients’ blood. For these reasons mentioned, the routine diagnostic determination of the subtypes of the monocytes (which are classical, intermediate, and nonclassical monocytes) already might be important today in order to be able to better assess the prognosis of the disease. In the near future, there may be drugs which are inhibitors of (nonclassical) monocytes in particular. If these drugs are available, flow cytometry will be an important tool for identifying MS patients who can then benefit from this new type of therapy. Up until now, it has been unclear if the presence of the nonclassical monocytes in MS patients is of a compensatory or causal nature.

## 5. Conclusions

This systematic review highlights the complex roles of monocyte subsets in MS pathogenesis. An in-depth analysis of both human and EAE studies revealed that immune cells play distinct roles in modulating inflammatory responses and neurodegenerative processes in MS. These findings underscore the dynamic nature of monocyte behavior, which encompasses the range of proinflammatory activation to anti-inflammatory responses, as well as the involvement of these cells in the initiation, progression, and resolution of MS. The findings of this review further suggest a potential role of monocyte subsets as biomarkers for monitoring MS disease activity and assessing the efficacy of therapeutic interventions. Alterations in monocyte populations during MS relapse and remission, coupled with changes in surface receptor expression, reveal the immunopathological mechanisms underlying MS progression. Therapeutic interventions targeting monocyte functions could add to the body of known immunomodulatory strategies to alleviate MS symptoms. Overall, this systematic review provides a comprehensive understanding of the roles of monocyte subsets in mitigating MS. Future research can leverage these findings to establish monocyte-mediated interventions for MS. These insights into the complexities of monocyte-mediated immune responses in MS offer a blueprint for tailored therapeutic interventions against this disease.

## Figures and Tables

**Table 1:** Details of the selected studies. The details of the selected studies, including the research design, sample size, EDSS criteria, and study conclusions, are listed to justify their inclusion in the study.

Study	Research Design	Research Subjects	MS Cases	Healthy Controls	Monocyte Definition	MS Sclerosis Duration	EDSS Criteria Reported by Authors	EDSS Score	Mean Age of MS Patients	Mean Age of Healthy Controls	Conclusion
Carstensen et al. [9]	Cohort study	Humans	CIS = 22 RRMS = 33 PMS = 6 RIS = 6 SC = 22	None	CD14 and CD16	2 years	McDonald criteria	CIS = 2 RRMS = 2.5 PMS = 3.0 RIS = N/A SC = N/A	CIS = 38 RRMS = 37 PMS = 52 RIS = 39.5 SC = 37	None	Incipient MS patients had reduced levels of nonclassical monocytes compared to SC and RIS patients. Notably, human endogenous retrovirus (HERV) expression was exhibited in nonclassical monocytes notwithstanding MS due to inflammatory activation.
Haschka et al. [10]	Cohort study	Humans	N = 65	N = 15	CD15^+^ neutrophils Classical monocytes Nonclassical monocytes	Active PMS—over 6 months PMSI—no progression over the last 6 months RRMSa—relapse within the last 3 months RRMSi—no relapse within the last 3 months	McDonald criteria	Baseline = ≤5.5 After treatment = Not indicated	MS = 44 years	HD = 44 years	Patients with inactive relapsing–remitting multiple sclerosis (RRMSi) had expanded volumes of classical and nonclassical monocytes, distinguishing between RRMSi and other types of MS.
Yang et al. [14]	Clinical trial	Humans	Not indicated	Not indicated	CD14 CD16	Not indicated	Not indicated	Not indicated	Not indicated	Not indicated	Unlike those in the control group, the monocytes in MS patients exhibited an elevated inflammatory profile with a high expression of CD40, CD86, CD64, and CCR2. Trichuris suis (TsSP) modulated the function and phenotype of macrophages, lowering the production and expression of inflammatory cytokines, thereby promoting an anti-inflammatory M2 signature.
Fischer et al. [15]	Clinical trial	Humans	N = 30	N = 30	CD14 CD16	Not indicated	McDonald criteria	RRMS = 2.54 SPMS = 6.12 PPMS = 5.5 Healthy controls = N/A	49.9	29.4	Classical CD14^++^CD16^−^ monocytes were found to be more abundant in MS patients than in healthy individuals, while nonclassical CD14^+^CD16^++^ monocytes were less frequent in MS patients. In addition, glucocorticoid treatment polarized monocytes into an anti-inflammatory phenotype, enabling their migration into the central nervous system and causing them to suppress pathogenic immune responses.
Khater et al. [16]	Cross-sectional study	Humans	N =22 patients in relapse N = 22 patients in remission	N = 44	CD14 CD16	3–6 years	McDonald criteria	Baseline = 2.74 ± 1.34 Results Classical = −0.202 Intermediate = 0.188 Nonclassical = 0.231	50 years	50 years	Patients with relapse and remission experienced an increase in all three monocyte subsets (classical, intermediate, and nonclassical). The increase in the monocyte subsets suggests their role in disease pathology since they are attributable to disease activity. These findings suggest a possible MS diagnostic tool and therapeutic target for MS.
Gjelstrup et al. [17]	Clinical trial	Humans	N = 40 patients	N = 20 healthy controls	CD14 CD16 CD40 CD163 CD192	11–33 months	McDonald criteria	Not indicated	Not indicated	Not indicated	Patients with MS had expanded nonclassical monocytes. The results indicate the relevance of monocytes, specifically nonclassical monocytes, in monitoring inflammatory diseases.
Kong et al. [18]	Clinical trial	Humans	N = aquaporin 4IgG-positive NMOSD patients N = 20 MS patients	N = 20 healthy controls	CD14 CD16	Not indicated	McDonald criteria	Baseline = 2.5 (average) Outcome = 2.13	Not indicated	Not indicated	An altered monocyte inflammatory response was indicated by elevated cell-surface molecules and a reciprocal dysregulation of anti-inflammatory and inflammatory cytokines. Notably, the monocytes from neuromyelitis optica spectrum disorder patients exhibited greater amounts of CD14^+^CD16^++^ nonclassical monocytes compared to the MS patients and healthy controls.
Waschbisch et al. [19]	Randomized clinical trial	Humans	RRMS patients (n = 40)	HD (n = 40)	CD14^+^ CD16^++^	Less than 2 months	McDonald criteria	HD = N/A RRMS = 1.5 NAT = 2.5 FTY = 2.0 IFN = 2.5	50 years	50 years	CD16^+^ monocytes were functional but reduced in the blood samples of MS patients. CD16^+^ monocytes were enriched in cerebrospinal fluid and were dominant among the CSF monocyte population. Conversely, MS patients with relapsing–remitting conditions exhibited an inverse CD16^+^ to CD16^−^ ratio.
Maleki et al. [20]	Experimental study	EAE mice	N = 20	N = 10	CD14 CD16 CD14^++^ CD16^+^	7 weeks	Not indicated	Not indicated	7 weeks	7 weeks	Muramyl dipeptide (MDP) treatment delayed EAE onset and decreased leucocyte infiltration in the central nervous system of mice. The results indicate the benefits of MDP in the progressive and early stages of EAE and can guide the development of MS medications and therapies.
Kapate et al. [21]	Experimental study	EAE mice	N = 3	N = 3	MHCII, CD80, CD86, and iNOS	48 h	Not indicated	Not indicated	6–11 weeks	6–11 weeks	The backpack-based intervention regulated the resident cells and infiltrated the myeloid cell compartments in the brain and the spinal cord, decreasing inflammation.
Meyer-Arndt et al. [22]	Experimental study	Humans	N = 4 RRMS donors	N = 5 allogeneic donors	CD14^+^ CD16^+^	Not indicated	Not indicated	Not indicated	30 years	47 years	Lipoic acid treatment inhibited monocytes from excreting the cytokines responsible for MS.
Adriani [23]	Experimental study	Humans	IFN-β–treated patients (n = 15) Treatment-naive patients (n = 10)	Healthy donors (HCs, n = 10)	CD14^+^ CD16^+^	9–18 months	McDonald criteria	Not indicated	Not indicated	Not indicated	During treatment with IFN-β, NOTCH2 expression on CD14^+^ monocytes and an increased release of proinflammatory monocytes predicted nADA development in MS patients.
Monteiro et al. [24]	Randomized clinical trial	Humans	N = 30 patients in remission N = 8 patients in relapse	Not indicated	CD14 CD16	2 years	McDonald criteria	Not indicated	51 years	NA	Plasmacytoid (pDC) and myeloid (mDC) dendritic cells decreased during remission but returned to normal values upon relapse. During both conditions, nonclassical monocytes decreased in frequency.

**Table 2:** Key papers in the field. The literature provides substantial insights into previous and contemporary findings on the role of monocytes in MS. The findings of these articles provide a blueprint for examining the research issues in depth and sufficiently bridging the knowledge gap.

Paper	Significant Findings
Carstensen et al. [9]	Incipient MS patients have reduced levels of nonclassical monocytes compared to patients with SC and RIS.
Haschka et al. [10]	Patients with inactive relapsing–remitting MS have expanded numbers of classical and nonclassical monocytes.
Fischer et al. [15]	Classical CD14^++^CD16^−^ monocytes are abundant in MS patients, but they have low levels of nonclassical CD14^+^CD16^++^ monocytes. These monocytes have anti-inflammatory effects.
Khater et al. [16]	Relapsing–remitting MS patients have an increase in all three monocyte subsets, revealing their role in disease pathology.
Gjelstrup et al. [17]	MS patients have abundant levels of nonclassical monocytes, and these have roles in monitoring inflammatory disease.
Kong et al. [18]	Elevated cell surface molecules and reciprocal dysregulation of the inflammatory and anti-inflammatory cytokines causes an altered monocyte inflammatory response.
Waschbisch et al. [19]	MS patients have reduced levels of CD16^+^ monocytes in their blood, but these monocytes are abundant in their CSF.
Maleki et al. [20]	Muramyl dipeptide (MDP) can delay EAE onset and prevent infiltration of the central nervous system in mice.
Monteiro et al. [24]	Monocyte levels are altered during remission and they normalize upon relapse.

**Table 3:** The 10 most important findings of the study. Critical insights into the roles of monocytes in MS have been reported. These findings will enable researchers to delve into this topic in depth.

The 10 Most Important Findings
The anti-inflammatory effects of monocytes due to the abundance of classical monocytes and the reduction in nonclassical monocytes in MS patients are highlighted. The variations in monocyte levels suggest a mechanism for tempering excessive inflammation to attenuate neuroinflammatory responses and achieve tissue homeostasis in MS patients.
The low expression of nonclassical monocytes in MS patients suggests their potential roles as biomarkers to monitor MS inflammation. The altered level of nonclassical monocytes in MS patients suggests their potential as disease biomarkers and their roles in the disease’s pathophysiology. This finding justifies the need to explore nonclassical monocytes as diagnostic indicators for MS, offering insights into its progression and developing a blueprint for tailored therapeutic interventions.
Monocytes exhibit altered inflammatory responses, characterized by increased cell surface molecules and the dysregulation of inflammatory and anti-inflammatory cytokines in MS patients. The surface molecules comprise CD40, CD86, and CD64, which initiate immune activation and promote interactions between monocytes and other immune cells. This response depicts a dysregulated immune milieu with an increased expression of cell surface molecules and a disrupted balance of inflammatory and anti-inflammatory cytokines. This dysregulation embodies the complex interplay between immune mediators during MS progression. These insights reveal the mechanism for disease progression and could enable researchers to develop targeted immunomodulatory therapies for MS management.
A tendency of monocyte levels to change during remission and normalize after relapse was observed, suggesting a dynamic immune response pattern associated with MS activity. The fluctuation in monocyte activity reveals their potential use in monitoring monocyte dynamics to determine disease status and progress. Researchers can use this information to identify the neuroinflammatory characteristics that trigger disease relapse and remission in MS patients.
MS patients with normal and altered motor functions were found to experience a significant decrease in surface receptors, indicative of potential immune cell signaling dysregulation, which may contribute to MS neurological impairment. The decreased surface receptors comprise CD169 and CD192, which modulate immune responses and cell-to-cell interactions. These findings highlight the broad scope of the impact of MS on immune cell functions beyond the central nervous system. Clinical researchers can use this information to establish a link between peripheral immune dysregulation and the clinical manifestations of MS.
The three monocyte subsets increase during relapse and remission of MS, indicating their involvement in the disease pathology. The dynamic involvement of monocytes in the disease pathogenesis reflects the fluctuating immune responses typical of the clinical course of the disease. This pattern emphasizes the distinct role of monocytes in MS inflammatory processes, identifying their critical role in the pathological mechanism of the disease relapse and remission cycle.
The elevated inflammatory profile, marked by high expression of CD40, CD86, CD64, and CCR2, reveals the role of monocytes in modulating MS pathological processes. This suggests that dysregulated monocyte activation via the increased expression of inflammatory markers contributes to MS pathology and offers the potential for targeted therapeutic interventions to modulate inflammation mediated by monocytes during MS.
Interventions, such as muramyl dipeptide (MDP), which slow disease progression and neuroinflammation in mice, have been reported. Clinical researchers could implement these findings to target specific immune pathways and develop therapeutic measures to delay or prevent MS onset or progression.
CD16^+^ monocytes are elevated in the cerebrospinal fluid and reduced in blood samples from MS patients, implying the potential migration of these cells from the circulatory system into the central nervous system as the disease progresses. These findings suggest the trafficking of immune cells across the blood–brain barrier, emphasizing the need to understand the mechanism by which monocytes infiltrate the central nervous system. Clinical researchers can use this information to establish therapeutic interventions that modulate neuroinflammatory responses.
The reduction in nonclassical monocytes in incipient MS patients compared to those with clinically isolated syndrome was confirmed, revealing the potential utility of these cells as diagnostic biomarkers. The observed behavior of nonclassical monocytes suggests their potential discriminatory role as diagnostic biomarkers to identify MS at early stages. These results suggest that clinical researchers could use nonclassical monocytes to distinguish between MS and other neurological conditions, leading to effective differential diagnosis.
